# Muscle Mass Moderates Metabolic Syndrome Risk Associated with Adiposity: A SHAP-Based Machine Learning Study

**DOI:** 10.3390/nu18091443

**Published:** 2026-04-30

**Authors:** Rodrigo Yáñez-Sepúlveda, Boryi A. Becerra-Patiño, Santiago Ramos Bermúdez, Rodrigo Olivares, Eduardo Guzmán-Muñoz, Yeny Concha-Cisternas, Daniel Rojas-Valverde, Carlos Abraham Herrera-Amante, Nicole Aguilera-Martínez, Camila Miño, José Francisco López-Gil

**Affiliations:** 1 Faculty Education and Humanities, Universidad Andres Bello, Viña del Mar 2520000, Chile; rodrigo.yanez.s@unab.cl; 2 Department of Sport Sciences, Faculty of Sport and Health Sciences, Fit Generation Research Institute, AD500 Andorra la Vella, Andorra; 3 Faculty of Physical Education, National Pedagogical University, Bogotá 480100, Colombia; babecerrap@pedagogica.edu.co; 4 Programa Licenciatura en Educación Física, Recreación y Deportes, Universidad Tecnológica del Chocó, Quibdó 274057, Colombia; santiago.ramos@ucaldas.edu.co; 5 Escuela de Ingeniería Informática, Universidad de Valparaíso, Valparaíso 2340000, Chile; rodrigo.olivares@uv.cl; 6 Escuela de Kinesiología, Facultad de Salud, Universidad Santo Tomás, Talca 3460000, Chile; eguzmanm@santotomas.cl (E.G.-M.); yenyconchaci@santotomas.cl (Y.C.-C.); 7 Escuela de Pedagogía en Educación Física, Facultad de Educación, Universidad Autónoma de Chile, Talca 3460000, Chile; 8 Vicerrectoría de Investigación e Innovación, Universidad Arturo Prat, Iquique 1100000, Chile; 9 Centro de Investigación, Desarrollo e Innovación en Salud y Deporte (CIDISAD), Escuela Ciencias del Movimiento Humano y Calidad Vida (CIEMHCAVI), Universidad Nacional de Costa Rica, Heredia 863000, Costa Rica; drojasv@hotmail.com; 10 Nutritional Assessment and Nutritional Care Laboratory (LECEN), Division of Health Sciences, Tonala University Center, University of Guadalajara, Tonalá 45425, Mexico; carlos.amante@academicos.udg.mx; 11 Escuela de Nutrición y Dietética, Facultad Ciencias de la Salud, Universidad Católica del Maule, Talca 3460000, Chile; naguilera@ucm.cl; 12 Universidad Internacional para el Desarrollo (UNINDE), 06011 Badajoz, Spain; camilamino501@gmail.com; 13 School of Medicine, Universidad Espíritu Santo, Samborondón 092301, Ecuador; 14 Vicerrectoría de Investigación y Postgrado, Universidad de Los Lagos, Osorno 5290000, Chile

**Keywords:** artificial intelligence, algorithms, health, obesity

## Abstract

**Background and Objective:** Previous studies have shown that muscle mass and visceral fat are interrelated and affect metabolic health. However, there is limited research exploring machine learning (ML) models that can help us understand the relationship between muscle mass and the risk of adiposity in the adult population. The objective of this study was to identify predictors of obesity on the basis of data from 13,663 adults assessed via body composition analysis via optimal and interpretable ML algorithms. **Methods:** A cross-sectional design was used to analyze data from 13,663 adults, comprising men (*n* = 6877) and women (*n* = 6786). The variables were obtained via 8-point multifrequency BIA under standardized clinical protocols with an Inbody^®^ Model 770 device validated for the adult population. To illustrate the interaction between body composition components, a probability heatmap was generated on the basis of the values predicted from the logistic model. The decision boundary was defined via the metabolic risk probability gradient, allowing visualization of the two-dimensional transition between low- and high-risk states. Statistical processing and figure generation were performed via Python software v.3.10. **Results:** The evaluation of the 10 algorithms demonstrated exceptional predictive performance, with the multilayer perceptron (MLP) standing out as the superior model in both sexes. The AUC-ROC was 0.981 for men and 0.993 for women, with F1 scores of 0.912 and 0.969, respectively. Overall, systematically higher accuracy was observed in the female cohort, exceeding 95% accuracy in most models. **Conclusions:** Muscle mass has been shown to act as a metabolic mediator, modulating and reducing the risk associated with visceral adiposity. It also concludes that the use of ML algorithms, specifically neural networks, is a good model for analyzing the risk associated with excess visceral fat.

## 1. Introduction

The scientific literature has shown that muscle mass and visceral fat are interrelated and influence metabolic health [1,2]. Thus, the assessment of adiposity factors reflects an interaction of multiple factors, including biological indicators; habits and lifestyles; and economic, health, and psychosocial determinants [3].

One study examined whether greater muscle mass is associated with lower mortality rates in older adults and concluded that relative muscle mass can be used to predict survival in these population groups [4]. A negative correlation has been reported between the muscle quality index (MQI) and insulin resistance (IR), as well as with type 2 diabetes mellitus, which manifests as a greater risk for the obese population [5,6].

High levels of adiposity, defined as a body mass index (BMI) greater than 30 kg/m^2^, have been linked to impaired glucose metabolism [7] and an increased risk of cardiovascular disease [8,9] and are also linked to an elevated mortality rate according to several prospective studies [10,11,12]. In this context, muscle mass has been described as a factor that modulates the associations among body mass index, obesity, and mortality [13], making it a factor that needs to be preserved and studied [14].

Other studies have shown that muscle mass and adipose tissue have different effects on carbohydrate metabolism [15], as well as on health risks resulting from the dual burden on physiological homeostasis [16]. Thus, the loss of muscle mass and increase in adipose tissue cause metabolic alterations and lead to duplicated functions in physiological homeostasis, with the visceral fat area (VFA) being associated with metabolically healthy obesity and with metabolically unhealthy nonobese individuals, reaffirming the need to identify obesity-related phenotypes across different population groups and longitudinal interventions [16].

Thus, a recent study by Gao et al. [17] revealed that a body composition (BC) characterized by high muscle mass and fat mass leads to glucose abnormalities (dysglycemia) and higher concentrations of cholesterol and/or triglycerides or lower levels of cholesterol and high-density lipoproteins (dyslipidemia), whereas higher levels of muscle mass and normal fat levels are associated with a lower risk of hyperglycemia. In this context, a review reveals that understanding the adipose-to-muscle ratio (AMR) as a more in-depth and clinical descriptor of the scope of obesity is necessary, allowing for informed decision-making within a broader framework [18].

Therefore, determining whether muscle mass can act as a moderator of obesity risk in adults is a research topic that needs to be addressed at this time. This study presents an innovative approach based on the use of machine learning (ML), drawing on artificial intelligence (AI) techniques such as SHapley Additive exPlanations (SHAP) to develop models for predicting obesity risk. These models have been developed for overweight adults to predict obesity risk and, thereby, combat health-related issues in the population [19]. This type of model has been used in medical prediction models [20]. A review study on various ML models aimed at investigating obesity revealed that a variety of methodologies are used to characterize and analyze obesity alongside other weight-related covariates [21], as well as the creation of a model that identifies the risk of obesity (ObeRisk) within an ML framework with high accuracy, precision, and sensitivity (~96.5%) [22]. Such applications of new tools, such as ML models with SHAP, could support clinical decision-making through greater data integration [23].

Scientific evidence has shown that muscle mass divided by the square of height is associated with greater insulin sensitivity and a lower metabolic risk [24]. However, to the best of our knowledge and according to a review of the scientific literature, there are knowledge gaps and gaps in current research on ML models that can be used to understand the relationship between muscle mass and the risk of adiposity in adults. Therefore, the aim of this study was to identify factors that may predict metabolic risk associated with adiposity via ML algorithms.

## 2. Materials and Methods

### 2.1. Study Design and Population

A cross-sectional analytical study was conducted using a database comprising a total of 13,663 adult subjects (men and women) who underwent BC analysis. The data was anonymized and derived from retrospective clinical records. Adult subjects aged 18–50 years with complete records were included. To preserve data integrity, exclusion criteria were applied, discarding records with missing values for the visceral fat level variable. Additionally, observations with biologically abnormal values, identified as extreme outliers through inspection of the interquartile range (IQR), were removed. Due to the retrospective nature of the dataset, no information on lifestyle factors (physical activity, dietary intake, tobacco use, alcohol consumption, or medication) was available. Consequently, no statistical adjustment for these potential confounders was performed in the models, and this limitation is further discussed in Section 4.1.

### 2.2. Anthropometric Measurements and Bioimpedance

The variables were obtained via 8-point multifrequency BIA under standardized clinical protocols with an Inbody^®^ Model 770 device validated for the adult population; the assessments were conducted at three research centers. Specifically, the InBody^®^ Model 770 (InBody Co., Ltd., Seoul, Republic of Korea) is an 8-point tactile electrode, segmental, multi-frequency bioelectrical impedance analyzer (Direct Segmental Multi-frequency BIA, DSM-BIA) that measures impedance at six frequencies (1, 5, 50, 250, 500, and 1000 kHz) across five body segments (right upper limb, left upper limb, trunk, right lower limb, and left lower limb). The manufacturer reports that the device relies on direct segmental impedance measurements and proprietary algorithms rather than on population-specific empirical prediction equations (e.g., Janssen, Kyle). All measurements were conducted under standardized conditions (morning, fasted state, after voiding, light clothing, and without metallic objects), following the manufacturer’s protocol [25].

The clinical evaluation protocol included a multidimensional characterization of the participants, covering demographic, anthropometric, metabolic, and advanced BC parameters [26]. Age, height, and body weight were recorded, from which body mass index (BMI) and the waist–hip ratio (WHR) was calculated as conventional anthropometric indicators. BC, including body fat percentage (%–kg) and skeletal muscle mass expressed in kilograms (%–kg), was determined via BIA. To assess central metabolic risk, both the visceral fat level and the visceral fat area (cm^2^) were quantified. Finally, biological and energy functional metrics, specifically the basal metabolic rate (BMR) and phase angle (°), were incorporated, providing a comprehensive view of the nutritional status and cellular integrity of the samples.

### 2.3. Statistical Analysis

An initial descriptive analysis was performed to characterize the sample, with continuous variables expressed as the mean and standard deviation (±). The normality of the data distribution was assessed via the Kolmogorov–Smirnov test and visual inspection of histograms and Q–Q plots. To compare anthropometric and BC characteristics between men and women, Student’s *t* test for independent samples was used for normally distributed variables, or the Mann–Whitney U test was used for those that did not meet the criteria for normality. The homogeneity of variances was verified via Levene’s test. All hypothesis tests were two-tailed, with a *p* value < 0.05 considered statistically significant. The assessment of diagnostic ability and the modeling of metabolic risk were addressed via an initial statistical approach.

First, receiver operating characteristic (ROC) curves were used to determine the discriminatory validity of body mass index (BMI), body fat percentage, and the interaction between muscle percentage and body fat percentage. The accuracy of each indicator was quantified via the area under the curve (AUC), and optimal cutoff points were identified via the Youden index (J), maximizing concurrent sensitivity and specificity. To subsequently examine the nonlinear dynamics of risk, binary logistic regression models were fitted, incorporating a multiplicative interaction term between adipose tissue and muscle mass (%Fat × %Muscle). To illustrate the interaction between BC components, a response surface map (probability heatmap) was generated on the basis of the predicted values from the logistic model. The decision boundary was defined via the gradient of metabolic risk probability, allowing visualization of the two-dimensional transition between low- and high-risk states. Statistical processing and figure generation were performed via Python v.3.10 (Scipy and Statsmodels libraries) [27].

### 2.4. ML Analysis

The computational implementation was based on the Python language (v.3.10) and used Pandas (v.2.1.4) and NumPy (v.1.26.4) for big data management and SciPy/Statsmodels (v.1.11.4) for inferential and interaction statistical modeling. The development of supervised learning algorithms and sample balancing via SMOTE were performed with Scikit-learn (v.1.3.2) and Imbalanced-learn (v.0.11.0), integrating an Explainable Artificial Intelligence (SHAP IA) framework via the SHAP library (v.0.44.0) to quantify the contributions of fat, muscle, and their interaction. Finally, high-resolution visualization of decision boundaries, ROC curves, and marginal effects was performed via Matplotlib (v.3.8.2) and Seaborn (v.0.13.1).

#### 2.4.1. Definition of the Outcome Variable

To train the classification algorithms, “High Metabolic Risk” was defined on the basis of the visceral adiposity phenotype, given its direct causal role in insulin resistance, type 2 diabetes, and cardiovascular disease, which is greater than that of subcutaneous fat.

#### 2.4.2. Classification of the Outcomes

The outcome was classified on the basis of a binary variable, with high risk (1) defined when the visceral fat level was ≥10—the standard clinical cutoff associated with metabolic dysfunction as established by the InBody^®^ 770 manufacturer specifications and validated in previous studies linking this threshold with metabolic syndrome components, insulin resistance, and cardiometabolic risk markers [28]. Low risk (0) was classified when the visceral fat level was <10.

#### 2.4.3. Feature Engineering and Balancing (SMOTE)

A nonlinear interaction term (% body fat × % muscle mass) was included, and a Z score transformation was applied. To correct for class imbalance, the synthetic minority oversampling technique (SMOTE) was used on the training set, ensuring optimal diagnostic sensitivity by artificially balancing the minority class within each sex-stratified cohort.

#### 2.4.4. Model Development and Cross-Validation

Ten supervised learning algorithms (random forest, GBM, SVM, and neural networks) were evaluated. A 10-fold cross-validation was implemented, where the model was trained and validated iteratively on different partitions of the sample to ensure the stability of the hyperparameters and mitigate overfitting. The ten algorithms were specifically selected to represent the main families of supervised classifiers—linear (Logistic Regression, Linear Discriminant Analysis), distance-based (K-Nearest Neighbors, Support Vector Machine with RBF kernel), tree-based ensembles (Random Forest, Gradient Boosting, AdaBoost, Decision Tree), probabilistic (Naive Bayes), and neural networks (Multilayer Perceptron)—in order to enable a fair and comprehensive comparative assessment. Hyperparameters were initialized with Scikit-learn default values and subsequently tuned through grid search embedded in the 10-fold stratified cross-validation procedure, selecting the configuration that maximized the mean AUC-ROC across validation folds. A fixed random seed (random_state = 42) was applied to all stochastic algorithms to ensure reproducibility. For distance- and gradient-based models (SVM, KNN, MLP, and Logistic Regression), features were standardized using a Z-score transformation (μ = 0, σ = 1). To prevent data leakage, the scaling parameters were fitted solely on the training partition and subsequently applied to transform the independent validation set.

#### 2.4.5. Neural Network Configuration

A multilayer perceptron (MLP) neural network was selected as the final architecture because it offered the best trade-off between predictive performance and computational efficiency across the validation folds and because it is particularly suited to capture the nonlinear interaction between skeletal muscle mass and body fat percentage that is central to this study. The following hyperparameters were used: two hidden layers with 50 and 100 neurons, the ReLU activation function, the Adam optimizer, and L2 regularization (Alpha = 0.0001), with a maximum of 1000 iterations. This configuration was selected after grid search over the number and size of hidden layers (tested configurations: [50], [100], [50,100], [100,50], [100,100]), activation functions (ReLU vs. tanh), and regularization strength (Alpha = 0.00001, 0.0001, 0.001), retaining the combination that maximized mean AUC-ROC in cross-validation.

#### 2.4.6. Performance Evaluation Metrics

For comprehensive diagnostic validation, model performance was quantified via the following metrics on the independent test set. Confusion matrices stratified by sex were generated to calculate true positives (TP), true negatives (TN), false positives (FP), and false negatives (FN). Accuracy was used to measure overall performance, and the F1 score was used as a metric for the balanced trade-off between precision and sensitivity, ensuring the model’s robustness across both classes. The error of each model was also evaluated. Discriminatory power was assessed via receiver operating characteristic (ROC) curve analysis. The area under the curve (AUC) was used as the definitive indicator of the model’s ability to distinguish between individuals with and without metabolic risk. Sensitivity (recall) and specificity were also analyzed to minimize diagnostic omissions of risk owing to excess visceral fat.

#### 2.4.7. Precision Imaging and Diagnostics (SHAP)

To ensure the interpretability and transparency of the predictive model, an XAI analysis was implemented using SHapley Additive exPlanations (SHAP) values [29,30]. Global importance plots (SHAP bar plots) and dependency scatter plots (SHAP summary plots) were generated to identify patterns of local and global influence, ensuring that the hierarchy of predictors was biologically consistent with the observed pathophysiology.

#### 2.4.8. Data Split and Independent Validation

To ensure model validity and avoid overfitting, the dataset was randomly split into 70% for training and 30% for independent validation. All the performance metrics, including the confusion matrix and the ROC curve, were calculated exclusively on this 30% of the unseen data, ensuring that the results reflect the algorithm’s actual ability to diagnose new clinical cases.

## 3. Results

Table 1 shows that the descriptive analysis of the study population (N = 13,663) revealed differences by sex in all the anthropometric and BC parameters assessed (*p* < 0.002). Men (*n* = 6877) had greater values for height, weight, and muscle mass, both in absolute terms and as percentages (42.23 ± 4.88% vs. 35.24 ± 4.51%), as well as a greater phase angle and basal metabolic rate. In contrast, the female cohort (*n* = 6786) presented a substantially greater body fat percentage (35.48 ± 8.34%) and, notably, a significantly greater level of visceral fat (12.75 ± 4.98 vs. 9.44 ± 4.53) despite having a lower average weight and BMI.

**Figure 1:**
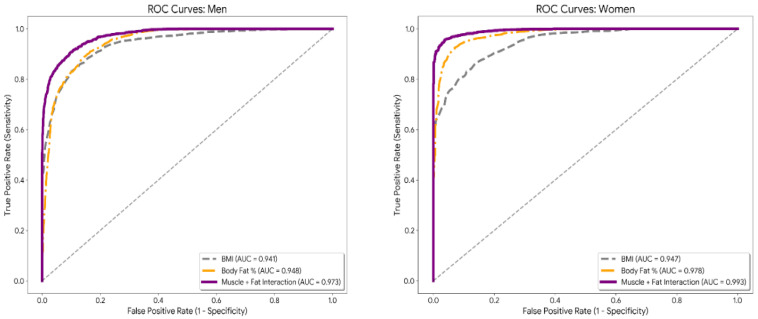
Comparison and cutoff points of ROC curves by sex.

Figure 1 shows that incorporating muscle–fat interactions resulted in greater diagnostic accuracy, with AUCs of 0.973 for men and 0.993 for women, significantly outperforming BMI and body fat percentage alone.

**Figure 2:**
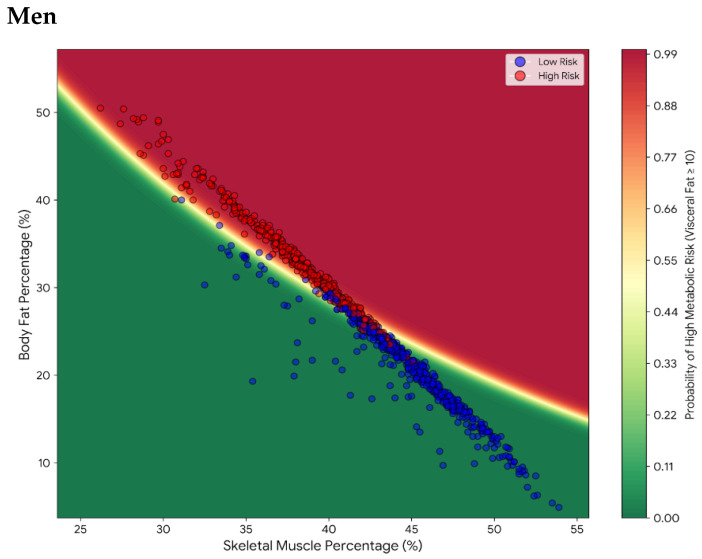

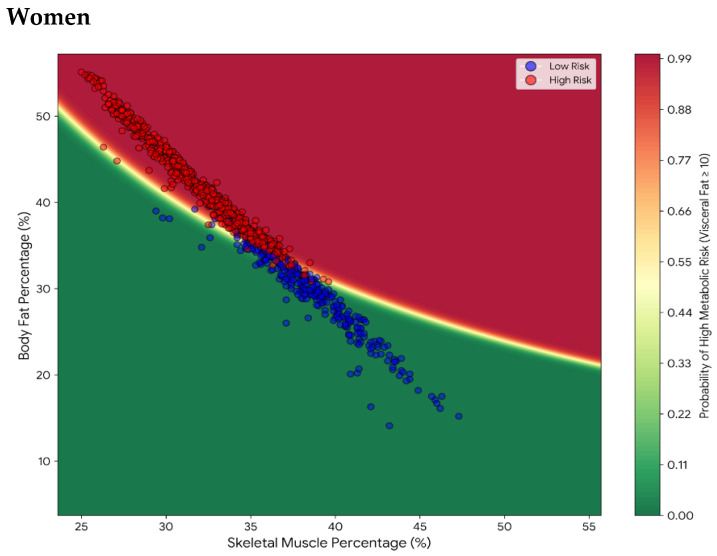
Metabolic risk maps: probability gradients of visceral adiposity by BC phenotype between men and women. Predicted probability of high visceral metabolic risk (visceral fat level ≥ 10) from the logistic model, displayed as a function of body fat percentage (x-axis) and skeletal muscle percentage (y-axis). Color scale: green (low probability) to red (high probability).

There is an inverse relationship: a greater percentage of muscle mass corresponds to a lower percentage of body fat, whereas lower muscle mass corresponds to greater body fat. There is also a concentration of red points (high risk) that coincides with the red zone of the predictive model, characterized by high levels of body fat and low muscle mass, whereas the blue points (low risk) coincide with the green zone and are characterized by low body fat and high muscle mass in the data for men. These findings revealed that the model used for men consistently classified metabolic risk on the basis of BC data (Figure 2). For women, metabolic risk does not depend solely on body fat but rather on the existing relationship between muscle mass and body fat. Thus, the model suggests that muscle mass serves as a protective mechanism against metabolic risk (Figure 2).

Figure 2 displays the predicted probability of high visceral metabolic risk as a function of body fat percentage and skeletal muscle percentage, stratified by sex. The color scale represents the probability gradient derived from the logistic model.

**Figure 3:**
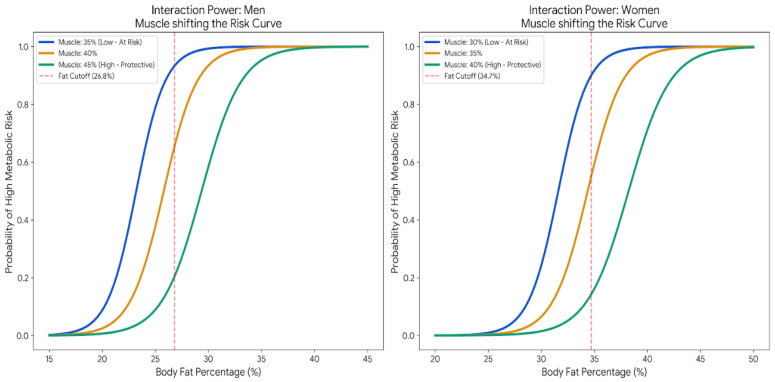
Sigmoid probability curves of visceral metabolic risk (visceral fat level ≥ 10) as a function of body fat percentage, plotted at three fixed levels of skeletal muscle percentage (low, medium, high) and stratified by sex.

The sigmoid probability curves in Figure 3 reveal a lateral shift in the risk threshold (risk shift) determined by muscle mass. In both sexes, reduced muscle mass (men: 35%; women: 30%) shifts the curve to the left, increasing metabolic vulnerability at levels of body fat considered normal. Conversely, high muscle mass (men: 45%; women: 40%) acts as a biological buffer, shifting the critical fat threshold to the right and conferring greater metabolic resilience. These findings confirm that the impact of adiposity is not constant but depends on tissue–tissue interactions, allowing for the identification of “normal-weight obesity” phenotypes that are invisible to conventional clinical screening.

Table 2 shows that the evaluation of the 10 algorithms demonstrated exceptional predictive performance, with the multilayer perceptron (MLP) standing out as the top-performing model for both genders. It achieved an AUC-ROC of 0.981 for men and 0.993 for women, with F1 scores of 0.912 and 0.969, respectively. Overall, a consistently higher accuracy was observed in the female cohort, exceeding 95% accuracy in most models.

In Table 3, the confusion matrices confirm the high effectiveness of the neural network: among men, there were 815 true negatives and 576 true positives, whereas among women, the accuracy was higher, with 689 true positives and only 22 false negatives.

**Figure 4:**
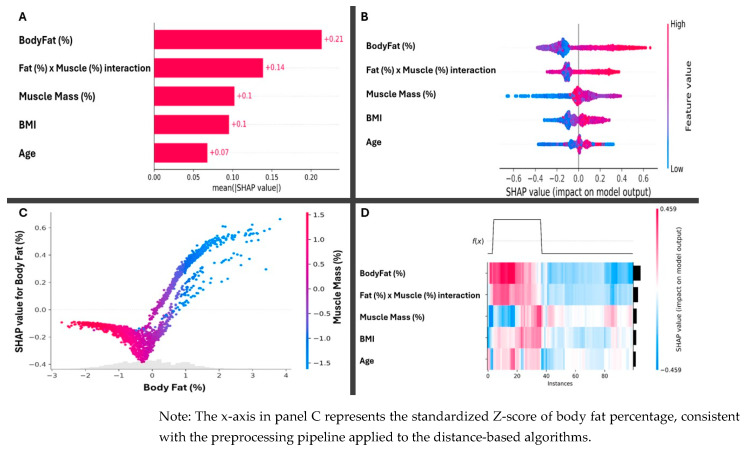
Decomposition of model interpretability via SHAP. (**A**) impact distribution analysis; (**B**) overall importance quantification; (**C**) dependency plot, the grey area is the actual distribution of the data in the percentage of fat; (**D**) heatmap and hierarchical clustering. BMI: body mass index.

As shown in Figure 4, the impact distribution analysis (Figure 4A) revealed that while body fat percentage was the primary risk factor, fat–muscle interaction exhibited a diagnostic polarity that defined the predictive trajectory across the entire cohort. When quantifying overall importance (Figure 4B), this interaction ranks as the second most significant factor in the model (SHAPmean = 0.14), surpassing the predictive power of BMI and age. The dependency plot (Figure 4C) illustrates the mechanics of this finding: at equivalent levels of adiposity, high muscle mass (indicated in fuchsia tones) exerts a buffering effect, shifting SHAP values toward protective ranges and revealing a critical inflection point at −0.5 SD. Finally, the heatmap and hierarchical clustering (Figure 4D) consolidate these data, allowing the identification of specific metabolic phenotypes where individual tissue architecture dictates the final probability of risk beyond conventional anthropometric thresholds.

## 4. Discussion

This study’s findings demonstrate a paradigm shift in the assessment of risk associated with excess visceral fat, showing through a multimodal explainability analysis that risk prediction is not simply a matter of classifying separate variables but rather the interaction of BC variables. Importantly, cross-sectional physiological differences (*p* < 0.001) underscore the importance of stratification by sex for subsequent predictive modeling via ML, highlighting distinct tissue and metabolic profiles between the two groups.

With the aim of identifying predictors of obesity in the adult population via ML models, which would help elucidate the relationship between muscle mass and the risk of adiposity in adults, data from 13,663 adults (49.7% women) assessed via BIA were analyzed. A visceral fat level greater than 10 was considered high risk (1), and a level less than 10 was considered low risk (0). The neural network (MLP) was the best predictive model for both sexes. These results are consistent with those of the present study, where the neural network (MLP) was also the superior model, achieving an AUC-ROC of 0.981 in men (F1 score = 0.912) and 0.993 in women (F1 score = 0.969). Other studies that have used ML algorithms indicate that these types of models have an advantage when analyzing the impact of obesity risk as a global health issue, where the decision tree method achieved 98.33% accuracy, followed by random forest (98.27%), leading to the conclusion that tree-based models are more effective at classifying obesity than traditional BMI approaches are [20].

A meta-analysis by Wang et al. [31] which included 81,358 participants and 11,969 deaths, revealed that a low skeletal muscle mass index was significantly associated with an increased risk of all-cause mortality, with a stronger association observed in individuals with a higher BMI. Moreover, SHAP model analyses revealed that global interaction analysis revealed that muscle mass can moderate the risk of adiposity, acting as a metabolic modulator such that higher levels of muscle mass reduce the risk associated with visceral adiposity and surpass the predictive capacity traditionally attributed to BMI. The present study confirms that muscle reserve is one of the most valuable indicators for assessing risk, with higher levels of muscle mass being considered a biological buffer, whereas the classification of underweight determined by BMI may fail to identify normal-weight obesity phenotypes in conventional screenings. Similarly, Moon et al. [32] reported higher all-cause mortality in adults under 65 years of age with low muscle mass, suggesting that higher SBM promotes metabolically healthy obesity, that is, obesity characterized by excessive body fat accumulation (BMI ≥ 30 kg/m^2^) without associated metabolic abnormalities [33]. Similar findings were reported by Liu et al. [34], who noted a significant increase in type 2 diabetes as the muscle-to-visceral fat ratio (SVR) quartiles decreased.

Furthermore, a higher total fat–muscle ratio (FMR) was reported to be associated with an increased risk of incident CVD and congestive heart failure, as shown by the UK Biobank, which followed 468,885 participants for 12.5 years [35], with an HR of 1.63 in men and 1.83 in women. Similarly, a low FMR was associated with higher fasting glucose (105 ± 27.5 vs. 96.8 ± 18.7 mg/dL, *p* < 0.001) and higher HbA1c (6.0 ± 0.8 vs. 5.8 ± 0.6%, *p* < 0.001) in 1060 older adults [36]. Total fat mass alone was associated with an elevated HR (1.20 for men, 1.25 for women) for CVD [37] and was identified by Hong et al. [38] as the predictor with the highest SHAP value, followed by BMI and subcutaneous adipose tissue.

The waist-to-hip ratio (WHR) has already been identified as a simple and effective screening tool [39], and analyses from the SHAP study identified it, along with the fat-free mass index (FFMI), as the most important predictive variable. Clinical thresholds of WHR > 0.92 and FFMI > 18.5 kg/m^2^ were established as predictors of unhealthy metabolic risk in the study by Deng et al. [40]. Sarcopenic obesity increased the risk of CVD by 95% (OR = 1.95, 95% CI: 1.62–2.36, *p* < 0.001) and CVD mortality by 64% (OR = 1.64, 95% CI: 1.15–2.34, *p* = 0.007) among 578,408 participants, as did atrial fibrillation (OR = 2.93, 95% CI: 2.23–3.65) and myocardial infarction (OR = 4.07, 95% CI: 1.31–12.63) [5]. With respect to the model’s predictive ability that LightGBM-SHAP achieved AUCs of 0.842 (MUNW vs. MHNW), 0.746 (MUO vs. MHO), and 0.968 (MUO vs. MHNW), with WHR and FFMI as the main predictors [40].

Some studies report higher all-cause mortality in adults with low muscle mass, suggesting that greater skeletal muscle mass promotes metabolically healthy obesity [41]. This finding is consistent with the results of the present study, which reported that increasing muscle mass could shift a person toward the lower-risk zone; however, there is a critical point (~40% muscle and ~30% fat) where the risk changes rapidly. For these reasons, further studies should continue implementing ML models to generalize models that help identify whether muscle mass moderates the risk of adiposity across different contexts, ages, and population groups. This is because some recent studies suggest that evaluating the body muscle–fat ratio (BMFR) could help identify high-risk phenotypes [42].

Moreover, some studies have shown that relative muscle mass is associated with lower insulin resistance due to increased glucose uptake and, consequently, a lower risk of developing type 2 diabetes [18,24]. In this context, the present study revealed that individuals with a greater proportion of skeletal muscle mass would also need to have high levels of adipose mass to reach the metabolic risk threshold than individuals with lower muscle mass. The findings of this study are related to the concept of metabolically healthy obesity, which is characterized as a phenotype of high adiposity without metabolic complications and is commonly associated with high muscle mass [43]. Other studies indicate that skeletal muscle mass serves as a primary pathway for eliminating postprandial glucose and acts as a regulator of insulin sensitivity to mitigate the negative effects of excess visceral fat [14,24]. In this regard, the SHAP dependency plot shown in Figure 4C quantitatively demonstrates that, regardless of body fat level, having higher muscle mass can systematically reduce the risk identified by the model used in this study.

### 4.1. Limitations and Strengths

Several limitations should be acknowledged when interpreting the results of this study. First, the cross-sectional design prevents the establishment of causal relationships between muscle mass, adiposity, and metabolic risk. Prospective studies are needed to validate whether increasing muscle mass over time leads to a reduction in metabolic risk, as suggested by the protective “buffer” effect observed in this analysis. Second, BC was assessed via multifrequency BIA. Although the InBody^®^ 770 device has been validated against criterion methods such as dual-energy X-ray absorptiometry (DEXA) and has demonstrated good agreement in adult populations [25], BIA remains a surrogate measure and may be subject to hydration status and other technical factors. Future studies incorporating gold-standard imaging techniques such as computed tomography or magnetic resonance imaging could provide more precise quantification of visceral and subcutaneous fat depots. Third, the definition of high metabolic risk was based on a visceral fat level ≥ 10, a clinically established cutoff associated with metabolic dysfunction. However, the absence of direct biochemical markers (such as fasting glucose, insulin, HbA1c, lipid profiles, or inflammatory cytokines) prevents the validation of this surrogate outcome against hard metabolic end points.

The integration of such biomarkers into future ML models would enable a more comprehensive assessment of the metabolic phenotype. Fourth, the study lacked data on lifestyle factors, including physical activity, dietary intake, and medication use, which are important confounders in the relationship between BC and metabolic health. Finally, the study population was derived from clinical records and may not be fully representative of the general adult population, potentially limiting the generalizability of the findings to other demographic or geographic groups.

We further acknowledge that the definition of metabolic risk used in this study is restrictive, as it relies exclusively on a visceral fat threshold without incorporating biochemical markers (fasting glucose, insulin, HbA1c, lipid profile) or inflammatory markers (hs-CRP, IL-6). This surrogate outcome captures only part of the metabolic phenotype, and the clinical relevance of our findings should therefore be interpreted within these boundaries. In addition, the retrospective origin of the dataset introduces potential selection bias—since only individuals seeking body composition assessment were included—and limits the generalizability of the results to the broader adult population.

Beyond these general limitations, several additional methodological aspects deserve explicit emphasis. First, the absence of adjustment for key behavioral and clinical confounders—physical activity, dietary patterns, tobacco use, alcohol consumption, medication (in particular antihypertensive, lipid-lowering, and glucose-lowering drugs), and socioeconomic status—constitutes a major constraint on the interpretation of our models. Individuals with greater skeletal muscle mass typically engage in more physical activity and follow healthier dietary patterns, both of which are independently protective against metabolic risk. Therefore, part of the apparent protective effect attributed to muscle mass in our models may reflect these unmeasured lifestyle factors rather than an independent causal contribution of muscle tissue. This unmeasured confounding should be explicitly acknowledged by readers when interpreting the clinical relevance of our findings.

Second, an important methodological caveat concerns the inherent non-independence of body composition compartments derived from bioelectrical impedance analysis. Fat mass, fat-free mass, and skeletal muscle mass are all estimated from the same raw impedance parameters (resistance and reactance at multiple frequencies) through proprietary regression equations that share common predictors. As a consequence, these variables are mathematically linked, and the strong negative correlation typically observed between fat mass and muscle mass (or their percentages) partially reflects this methodological dependence rather than an entirely independent biological signal. Moreover, as emphasized by Lagacé et al. (2019) [44], expressing body composition in percentages introduces a mathematical constraint (%fat + %fat-free mass ≈ 100%), which by construction produces near-perfect inverse correlations between these variables and may inflate the apparent strength of muscle-fat interactions. The inverse linear relationship shown in Figure 2 should therefore be interpreted with caution, as part of its linearity reflects this methodological constraint. To mitigate this concern, a complementary analysis using absolute values (kg) has been included as Supplementary Figure S1, which confirms that the muscle-fat interaction pattern is preserved while the apparent linearity is attenuated. Reference methods such as DEXA, computed tomography, or magnetic resonance imaging, which provide compartment-specific measurements, would be required to fully disentangle the biological interaction between tissues from the statistical artifact inherent to BIA [45].

Third, recent evidence by Lagacé et al. [46] based on NHANES data has shown that, counterintuitively, greater fat-free mass may be associated with increased odds of metabolic syndrome when expressed in absolute terms, challenging the straightforward interpretation that more muscle mass is universally protective. These findings highlight the complexity of muscle-fat interactions and the risk of circularity inherent to body composition variables derived from BIA, and they reinforce the need for prospective interventional studies to determine whether increases in skeletal muscle mass truly translate into improved metabolic outcomes.

Fourth, although 10-fold cross-validation and an independent 30% test partition were applied to mitigate overfitting, the absence of external validation on an independent cohort constitutes a relevant limitation. High performance metrics obtained on internal partitions may overestimate real-world generalizability. Moreover, while SHAP values provide local and global interpretability of individual predictions, their practical translation into clinically actionable decisions has not been empirically tested in prospective clinical settings. External validation in independent, prospectively collected cohorts—including biochemical outcomes—is required before the models can be considered for clinical deployment.

Despite these limitations, this study has notable strengths, including the large sample size (*n* = 13,663), rigorous sex-stratified analyses, and the use of an independent test set to evaluate model generalizability. The implementation of a comprehensive ML framework with 10 algorithms and the integration of SHAP analysis for model interpretability represent methodological advances that enhance both the predictive accuracy and the clinical utility of the findings. By explicitly quantifying the interaction between muscle mass and fat mass and demonstrating its superior predictive capacity relative to conventional metrics, this study provides a robust foundation for future research aimed at refining metabolic risk assessment.

### 4.2. Practical Clinical and Public Health Applications

The present findings may have potential clinical and public health implications, although any translational application must be considered preliminary and hypothesis-generating. By suggesting that muscle mass may modulate adiposity-related metabolic risk in a nonlinear fashion, this study provides a conceptual framework that could inform future research on dynamic, compositional assessments of body tissue architecture that go beyond static anthropometric thresholds. The probability heatmaps and the sigmoid curves presented here could, in principle, be further developed into clinical decision support tools; however, before any clinical deployment, prospective longitudinal validation and external validation in independent cohorts are required. Similarly, the tentative risk classification system suggested by the hierarchical clustering analysis (Figure 4D) should be regarded as an exploratory framework for future research rather than as a ready-to-use clinical criterion. Only after rigorous validation against hard metabolic and cardiovascular end-points could such an approach contribute to the emerging paradigm of precision medicine [23,47].

The following classification according to age and sex is recommended:


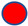
 High body fat/low muscle mass = high risk


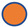
 Low body fat/low muscle mass = moderate risk


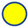
 High body fat/high muscle mass = low risk


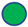
 Low body fat/high muscle mass = minimal risk

## 5. Conclusions

This study suggests that skeletal muscle mass may act as a metabolic modulator of the risk associated with visceral adiposity and that explainable machine learning algorithms, particularly multilayer perceptron neural networks, can be useful tools to capture the nonlinear interaction between muscle and adipose tissue. A dynamic and nonlinear relationship was observed that could complement, rather than replace, traditional anthropometric metrics for risk stratification. However, these findings should be interpreted with caution in light of the cross-sectional design, the surrogate nature of the outcome (visceral fat level ≥ 10, without biochemical metabolic markers), the inherent non-independence of body composition compartments derived from BIA, the absence of adjustment for key lifestyle confounders, and the lack of external validation. Therefore, the results of this study should be regarded as hypothesis-generating and as a methodological foundation for future research. Prospective longitudinal studies using reference methods for body composition assessment (DEXA, MRI), incorporating biochemical metabolic outcomes, adjusting for behavioral and pharmacological confounders, and including external validation cohorts, are required before the proposed framework can be translated into clinical practice or inform public health strategies.

## Figures and Tables

**Table 1:** Study population characteristics.

Variable	Men (*n* = 6877)	Women (*n* = 6786)	*p* Value
Age (years)	31.40 ± 12.13	27.44 ± 11.56	<0.001
Height (cm)	172.87 ± 9.37	159.77 ± 6.78	<0.001
Weight (kg)	82.40 ± 15.76	67.20 ± 15.08	<0.001
BMI (kg/m^2^)	27.45 ± 4.42	26.29 ± 5.63	<0.001
Body Fat Percentage (%)	25.46 ± 8.35	35.48 ± 8.34	<0.001
Skeletal Muscle Mass (kg)	34.49 ± 5.92	23.24 ± 3.79	<0.001
Skeletal Muscle Percentage (%)	42.23 ± 4.88	35.24 ± 4.51	<0.001
Visceral Fat Level	9.44 ± 4.53	12.75 ± 4.98	<0.001
Visceral Fat Area (cm^2^)	79.50 ± 42.87	83.27 ± 32.28	0.002
Waist-Hip Ratio	0.93 ± 0.09	0.92 ± 0.08	<0.001
Basal Metabolic Rate (kcal)	1681.91 ± 211.66	1286.41 ± 135.44	<0.001
Phase Angle (°)	6.35 ± 0.62	5.36 ± 0.59	<0.001

Note: cm: centimeters; kg: kilograms; m: meters; %: percentage; kcal: kilocalories.

**Table 2:** Performance of trained algorithms.

Sex	Algorithm	Accuracy	Error	Precision	Recall	F1 Score	AUC-ROC
Men	Neural Network	0.926	0.074	0.917	0.907	0.912	0.981
	Gradient Boosting	0.923	0.077	0.911	0.907	0.909	0.981
	Random Forest	0.922	0.078	0.915	0.899	0.907	0.979
	Logistic Regression	0.919	0.081	0.911	0.898	0.904	0.979
	K-Nearest Neighbors	0.925	0.075	0.916	0.907	0.911	0.975
	SVM (RBF)	0.921	0.079	0.911	0.902	0.907	0.973
	Linear Discriminant	0.916	0.084	0.928	0.869	0.898	0.978
	AdaBoost	0.914	0.086	0.913	0.880	0.897	0.978
	Naive Bayes	0.897	0.103	0.870	0.888	0.879	0.968
	Decision Tree	0.910	0.090	0.891	0.898	0.894	0.908
Women	Neural Network	0.956	0.044	0.970	0.969	0.969	0.993
	Logistic Regression	0.952	0.048	0.964	0.969	0.966	0.993
	AdaBoost	0.953	0.047	0.966	0.969	0.967	0.993
	SVM (RBF)	0.957	0.043	0.975	0.964	0.970	0.992
	Gradient Boosting	0.954	0.046	0.968	0.967	0.968	0.992
	Random Forest	0.951	0.049	0.966	0.966	0.966	0.989
	Naive Bayes	0.947	0.053	0.986	0.938	0.962	0.989
	Linear Discriminant	0.915	0.085	0.900	0.990	0.943	0.989
	K-Nearest Neighbors	0.953	0.047	0.962	0.973	0.967	0.981
	Decision Tree	0.941	0.059	0.960	0.957	0.958	0.930

Note: SVM: support vector machine; RBF: radial basis function.

**Table 3:** Confusion matrix: Neural network predictive validation for men and women.

Sex	Real Condition	Predicted: Low Risk	Predicted: High Risk
Men	Low Risk	815 (TN)	52 (FP)
	High Risk	59 (FN)	576 (TP)
Women	Low Risk	253 (TN)	21 (FP)
	High Risk	22 (FN)	689 (TP)

## Data Availability

Data are contained within the article.

## Supplementary Materials

The following supporting information can be downloaded at: https://www.mdpi.com/article/10.3390/nu18091443/s1.
