# Supplementary material for: Muscle Mass Moderates Metabolic Syndrome Risk Associated with Adiposity: A SHAP-Based Machine Learning Study
Source: Nutrients. 2026 Apr 30;18(9):1443. doi: 10.3390/nu18091443 (PMC13165194; doi:10.3390/nu18091443)
Supplement: Supplementary file 1 [file nutrients-18-01443-s001.zip › nutrients-4278052-supplementary.pdf]

nutrients

MDPI

Supplementary Materials

S1. Hyperparameter Configuration by Algorithm

Algorithm	Engine/Library	Key Hyperparameters (Configuration)
**Neural Network**	MLPClassifier	Hidden Layers: (100, 50), Activation: 'ReLU', Solver: 'Adam', Alpha: 0.0001, Max Iter: 1000.
**Gradient Boosting**	GBM	n_estimators: 100, learning_rate: 0.1, max_depth: 3, loss: 'log_loss'.
**Random Forest**	RandomForest	n_estimators: 100, criterion: 'gini', max_features: 'sqrt', bootstrap: True.
**Logistic Regression**	Logit	Penalty: 'l2', C: 1.0, Solver: 'lbfgs', Max Iter: 1000.
**AdaBoost**	AdaBoost	Base Estimator: DecisionTree, n_estimators: 50, learning_rate: 1.0.
**SVM (RBF)**	SVC	Kernel: 'rbf', C: 1.0, Gamma: 'scale', Probability: True.
**K-Nearest Neighbors**	KNN	n_neighbors: 5, Weights: 'uniform', Metric: 'minkowski' (p=2).
**Linear Discriminant**	LDA	Solver: 'svd', Shrinkage: None, Store Covariance: False.
**Naive Bayes**	GaussianNB	Var Smoothing: 1e-09 (Standard Gaussian Distribution).
**Decision Tree**	CART	Criterion: 'gini', Splitter: 'best', min_samples_split: 2.

Technical Implementation Note

Reproducibility: A fixed seed (random_state=42) was applied to all stochastic algorithms to ensure consistency across experimental iterations.

Data normalization: For distance-based and gradient-based models (SVM, KNN, MLP, and logistic regression), features were standardized via Z score transformation (\mu=0, \sigma=1) to prevent scale-related bias.

Architecture (Neural Network): The multilayer perceptron (MLP) was designed with a dual hidden-layer architecture to capture the complex, nonlinear interaction between skeletal muscle mass and total body fat percentage.

S2. Metabolic risk maps: probability gradients of visceral adiposity by BC phenotype between men and women.

 https://doi.org/10.3390/nu18091443

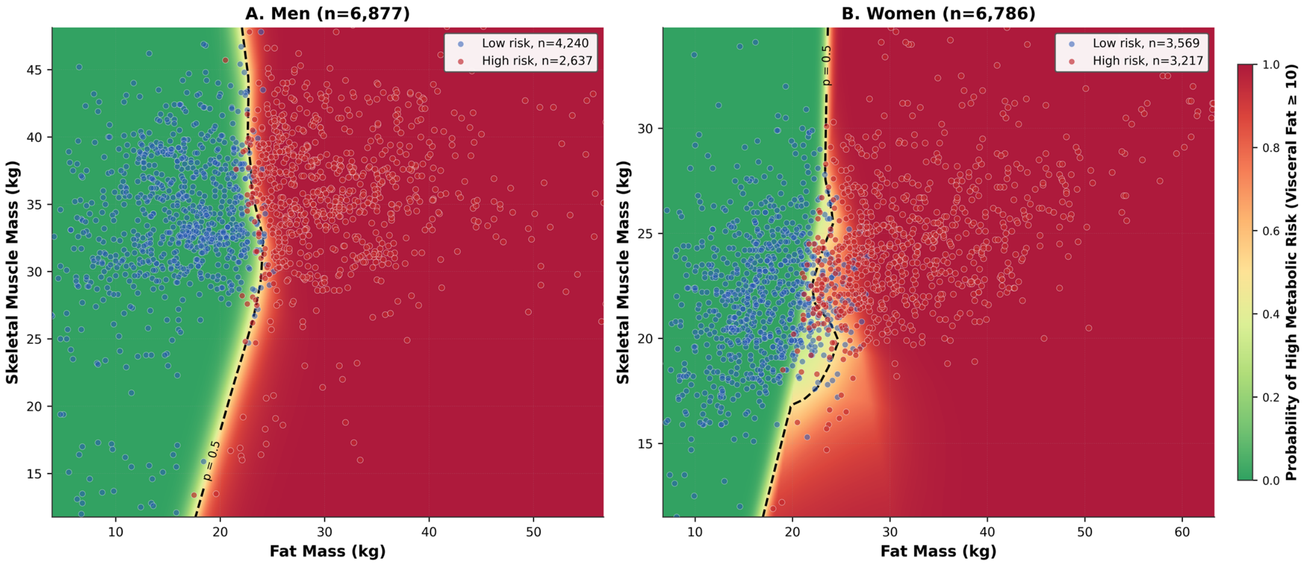

Figure S1. Probability heatmaps of high visceral metabolic risk as a function of absolute fat mass (x-axis, kg) and absolute skeletal muscle mass (y-axis, kg), stratified by sex. (A) Men (n = 6,877); (B) Women (n = 6,786). The colour scale represents the predicted probability of high metabolic risk from a multilayer perceptron (MLP) with the same architecture used in the main manuscript (two hidden layers, 50 and 100 neurons, ReLU, Adam, L2 α = 0.0001). The dashed black line marks the decision boundary at p = 0.5. Blue dots represent randomly sampled low-risk individuals; red dots represent high-risk individuals. High metabolic risk was defined as visceral fat level ≥ 10 (n = 8,288, InBody® VFL available) or visceral fat area ≥ 100 cm^2 (Level 10) (n = 5,375, InBody® VFA available), which are equivalent clinical cutoffs. The decision boundary in both panels is clearly non-vertical and shifts rightward with increasing skeletal muscle mass, indicating that higher muscle mass raises the fat-mass threshold at which metabolic risk becomes high — thus confirming the muscle-fat interaction dynamics reported in Figure 2 of the main manuscript (which used percentages), now demonstrated in absolute units.

S2.1. Key findings

S2.1.1. The inverse linearity observed in Figure 2 (%) is largely a methodological artefact

The Pearson correlation between body fat percentage (%BF) and skeletal muscle percentage (%SMM) is near-perfect and negative in both sexes (r = −0.986 in men, r = −0.988 in women, r = −0.986 in the overall sample), as expected from the mathematical constraint that the two percentages are near-complementary. However, when the same compartments are expressed as absolute values (fat mass in kg and skeletal muscle mass in kg), the correlation drops dramatically to +0.272 in men, +0.526 in women, and only +0.127 in the overall sample (Table S1). Spearman rank correlations show the same pattern. This demonstrates that the vast majority of the apparent inverse linearity visible in the main Figure 2 is an artefact of the unit of expression rather than a genuine biological coupling between the two tissues.

Table S1. Pearson and Spearman correlations between fat mass and skeletal muscle mass (percentage vs. kg).

Group	N	Pearson %	Pearson kg	Spearman %	Spearman kg
Men	6,877	−0.986	+0.272	−0.988	+0.247
Women	6,786	−0.988	+0.526	−0.989	+0.484
**Overall**	**13,663**	**−0.986**	**+0.127**	**−0.985**	**+0.114**

Note. Correlations are computed in the full analytical sample (N = 13,663). All correlations in percentage units are significant at $p < 0.001$ due to the mathematical dependency between complementary percentages.

S2.1.2. The muscle-fat interaction dynamics are preserved in kg

Figure S2 confirms that the muscle-fat interaction dynamics observed in the main Figure 2 are fully preserved when body composition is expressed in absolute units. In both men and women, the decision boundary at $p = 0.5$ shifts rightward as skeletal muscle mass increases, indicating that individuals with greater muscle mass tolerate a higher absolute amount of fat mass before reaching the high-risk zone. Quantitatively, in men the fat-mass threshold for high metabolic risk shifts from approximately 18 kg at low muscle mass (~15 kg SMM) to approximately 24 kg at high muscle mass (~45 kg SMM); in women, the threshold shifts from approximately 17 kg to approximately 25 kg across the observed muscle mass range. This dynamic modulatory role of skeletal muscle mass—the core biological finding of the manuscript—is therefore preserved in absolute units and is not an artefact of percentage-based representation.

S2.1.3. Predictive performance is consistent across parameterisations

As a further sensitivity check, we compared the cross-validated AUC-ROC of a multilayer perceptron using the two fat and muscle percentages versus the same two compartments expressed in kilograms (Table S2). Both parameterisations yielded excellent discrimination, with the kg-based model showing slightly higher AUC values (0.996 ± 0.001 in men and 0.984 ± 0.007 in women) than the percentage-based model (0.975 ± 0.009 in men and 0.975 ± 0.008 in women). This indicates that the predictive information carried by fat and muscle compartments is fully preserved—and even slightly enhanced—when the variables are expressed in absolute units, and rules out the possibility that the main results of the manuscript depend on the unit of expression.

Table S2. Cross-validated AUC-ROC for logistic regression using fat and muscle compartments in percentage vs. kg.

Group	N	AUC (% features)	AUC (kg features)
Men	6,877	0.975 ± 0.009	0.996 ± 0.001
Women	6,786	0.975 ± 0.008	0.984 ± 0.007

Note. Values are the mean ± standard deviation of the AUC-ROC across 5-fold stratified cross-validation using a multilayer perceptron (MLP) with the same architecture as in the main manuscript (two hidden layers with 50 and 100 neurons, ReLU activation, Adam optimizer, L2 α = 0.0001). Features were standardised prior to training. High metabolic risk was defined as VFL ≥ 10 or, where VFL was not available, as VFA ≥ 100 cm^2 (equivalent clinical cutoffs).
